# Stac3 Inhibits Myoblast Differentiation into Myotubes

**DOI:** 10.1371/journal.pone.0095926

**Published:** 2014-04-30

**Authors:** Xiaomei Ge, Yafei Zhang, Sungwon Park, Xiaofei Cong, David E. Gerrard, Honglin Jiang

**Affiliations:** Department of Animal and Poultry Sciences, Virginia Tech, Blacksburg, Virginia, United States of America; West Virginia University School of Medicine, United States of America

## Abstract

The functionally undefined Stac3 gene, predicted to encode a SH3 domain- and C1 domain-containing protein, was recently found to be specifically expressed in skeletal muscle and essential to normal skeletal muscle development and contraction. In this study we determined the potential role of Stac3 in myoblast proliferation and differentiation, two important steps of muscle development. Neither siRNA-mediated Stac3 knockdown nor plasmid-mediated Stac3 overexpression affected the proliferation of C2C12 myoblasts. Stac3 knockdown promoted the differentiation of C2C12 myoblasts into myotubes as evidenced by increased fusion index, increased number of nuclei per myotube, and increased mRNA and protein expression of myogenic markers including myogenin and myosin heavy chain. In contrast, Stac3 overexpression inhibited the differentiation of C2C12 myoblasts into myotubes as evidenced by decreased fusion index, decreased number of nuclei per myotube, and decreased mRNA and protein expression of myogenic markers. Compared to wild-type myoblasts, myoblasts from Stac3 knockout mouse embryos showed accelerated differentiation into myotubes in culture as evidenced by increased fusion index, increased number of nuclei per myotube, and increased mRNA expression of myogenic markers. Collectively, these data suggest an inhibitory role of endogenous Stac3 in myoblast differentiation. Myogenesis is a tightly controlled program; myofibers formed from prematurely differentiated myoblasts are dysfunctional. Thus, Stac3 may play a role in preventing precocious myoblast differentiation during skeletal muscle development.

## Introduction

Skeletal muscle is composed of multinucleated cells called myofibers, which are formed from the fusion of myoblasts. The process of formation of myofibers from muscle progenitor cells is called myogenesis, and this process is controlled by a set of transcription factors, including Myf5, Mrf4, MyoD, and myogenin (MyoG). While Myf5 and MyoD determine the myogenic lineage of muscle progenitor cells [1]–[3], MyoG and Mrf4 drive the terminal differentiation and fusion of myoblasts into myotubes, the developing myofibers [4]–[7]. Skeletal muscle plays important roles in initiating movements, supporting respiration, and maintaining homeostasis; loss of skeletal muscle mass or function is associated with ageing and diseases such as cancer and diabetes [8], [9]. Skeletal muscle in meat-producing animals is a tissue of major economic importance. Therefore, identification of new factors and mechanisms that control skeletal muscle development and physiology has significant implications for improving human health and animal productivity.

The functionally undefined Stac3 gene appears to be a new factor regulating skeletal muscle development and function. This gene is predicted to encode a protein containing two Src homology three (SH3) domains and a cysteine rich (C1) domain [10]. The Stac3 gene is specifically expressed in skeletal muscle [10]–[14]. Stac3 knockout in mice caused perinatal death and marked abnormalities in skeletal muscle [11], [13]. In zebrafish, morpholino-mediated Stac3 knockdown resulted in defective myofibrillar protein assembly [14]; absence of Stac3 expression due to a missense mutation in a splice donor site or morpholino-mediated Stac3 knockdown caused the fish to be immobile or shiver [12]. It was proposed that Stac3 plays a role in excitation-contraction coupling through interaction with the dihydropyridine receptor and the ryanodine receptor [11], [12].

The function of Stac3 has also been studied in C2C12 myoblasts [14]. Bower and colleagues observed that Stac3 knockdown prevented C2C12 myoblasts from fusing into myotubes and concluded that Stac3 is essential for myoblast differentiation and myotube formation [14]. However, an essential role of Stac3 in myoblast differentiation does not appear to be supported by the facts that Stac3 knockdown or mutation did not prevent the formation of myofibers in zebrafish [12], [14] and that Stac3 knockout did not block the formation of myofibers in mice [11], [13].

In this study, we examined the role of Stac3 in myoblast differentiation and myotube formation by determining the effects of Stac3 knockdown, overexpression, and knockout on myoblast differentiation and fusion into myotubes in both C2C12 myoblast line and mouse embryonic myoblasts. Our results suggest an inhibitory role of Stac3 in myoblast differentiation and myotube formation.

## Experimental Procedures

### Culture of C2C12 cells

C2C12 cells (ATCC, Manassas, VA) were expanded in growth medium composed of Dulbecco's Modified Eagle Medium (DMEM), 10% fetal bovine serum (FBS), and 1% Antibiotic-Antimycotic (ABAM) at 37 °C under 5% CO_2_. Differentiation was induced by replacing the growth medium with differentiation medium consisting of DMEM, 2% horse serum, and 1% ABAM. Cell culture media and supplements were purchased from Mediatech Inc. (Manassas, VA) unless otherwise specified.

### Stac3 knockdown

C2C12 cells were plated in 24-well plates and grown to approximately 70% confluence. Cells in each well were transfected with Stac3 siRNAs (MSS239387, MSS239388, MSS239389 from Invitrogen, Carlsbad, CA), in combination of three (10 nM per siRNA) or separately (30 nM). Control cells were transfected with 30 nM scrambled siRNA (Invitrogen). The transfection reagent was Lipofectamine 2000 (Invitrogen). Since an appropriate Stac3 antibody was not available, knockdown efficiency was estimated by quantitative RT-PCR of Stac3 mRNA.

### Stac3 overexpression

Stac3 cDNA was amplified from mouse skeletal muscle total RNA and linked to a Flag tag at the 3′ end via PCR using primers 5′-GAGGATCCGGGGCCCAATCTCTTGTAA-3′ and 5′-TCTCTAGACTACTTGTCATCGTCGTCCTTGTAATCGTAAATCTCCTCC-3′. The Stac3-Flag fusion cDNA was cloned into the pcDNA3.1-hygro (+) vector (Invitrogen) at the BamHI and XbaI sites to generate the plasmid pcDNA3.1/Stac3-Flag. To overexpress Stac3 in C2C12 cells, the cells at 70% confluence were transfected with 0.5 µg of pcDNA3.1/Stac3-Flag using Lipofectamine 2000 as the transfection reagent. Transfection of an equivalent amount of pcDNA3.1-hygro (+) plasmid served as a control. Overexpression of Stac3 was confirmed by quantitative RT-PCR of Stac3 mRNA and western blot analysis of Stac3-Flag fusion protein using an anti-Flag antibody (see details below).

### Stac3 mutant mice

Generation, breeding, and genotyping of Stac3 mutant mice were described in detail in a previous report [13]. Mice were housed on a timed 12 h light/dark schedule with free access to standard rodent diet and water. All procedures involving animals were approved by the Virginia Tech Institutional Animal Care and Use Committee.

### Isolation and culture of myoblasts from mouse embryos

Mouse embryonic myoblasts were isolated as described [15], with minor modifications. Briefly, limb muscles from embryonic day 17.5 (E17.5) embryos were dissected and digested in a solution consisting of 1.5 U/ml collagenase (Roche, Indianapolis, IN), 2.4 U/ml dispase (Roche), and 2.5 mM CaCl_2_ at 37 °C for 30 minutes. The released cells were collected by centrifugation and resuspended in Ham's F-10 medium supplemented with 20% FBS, 2.5 ng/ml basic fibroblast growth factor (Promega, Madison, WI), and 1% ABAM. The cells were allowed to attach to the plates for 2 h at 37 °C. The medium containing the unattached cells was transferred to collagen I-coated dishes (BD Biosciences, San Jose, CA). When the cells reached 50% confluence, they were subjected to 3 to 5 times of 20-min preplating or until 70% of them were estimated to be myoblasts [16]. To induce differentiation, cells at 80% confluence were switched to DMEM medium supplemented with 2% horse serum and 1% ABAM.

### Quantitative RT-PCR

Total RNA was isolated using TRI reagent according to the manufacturer's instructions (Molecular Research Center, Cincinnati, OH). Quantitative RT-PCR was performed essentially as described previously [13]. Stac3, myogenin (Myog), myosin heavy chain 3 (Myh3), myoglobin (Mb), troponin T type 1 (Tnnt1), troponin T type 3 (Tnnt3), and creatine kinase, muscle (Ckm) mRNAs were quantified by PCR using primer pairs 5′-TACAGCGACCAACAGTACGC-3′ and 5′-TCTGCATTGTTTCCATCCTG-3′; 5′-CGGCTGCCTAAAGTGGAGAT-3′ and 5′-AGGCCTGTAGGCGCTCAA-3′; 5′-CGCAGAATCGCAAGTCAATA-3′ and 5′-ATATCTTCTGCCCTGCACCA-3′; 5′-ATGTGAGGGCCAGAGAAAGG-3′ and 5′-TCCAGGTACTTGACCGGGAT-3′; 5′-AAACCCAGCCGTCCTGTG-3′ and 5′-TCATCTCCCGACCAGTCTGT-3′; 5′-GCCCAAGAGGAAGAAGTCCA-3′ and 5′-TAGCTGCTGTAGTTGGCACC-3′; and 5′-CGCAGCATCAAGGGTTACAC-3′ and 5′-AGGTGCTCGTTCCACATGAA-3′, respectively. mRNA abundance was calculated as relative to 18S rRNA, which was quantified by RT-PCR using primers 5′-TTAAGAGGGACGGCCGGGGG-3′ and 5′-CTCTGGTCCGTCTTGCGCCG-3′. Based on the Ct values, expression of 18S rRNA was not different (*P*>0.1) between the conditions used in this study.

### Immunocytochemistry

Cells were fixed with 4% paraformaldehyde in phosphate buffered saline (PBS) for 15 min and permeated with 0.25% Triton X-100 for 10 min at room temperature. Cells were then blocked with 1% bovine serum albumin (BSA) in PBST (PBS + 0.05% Tween-20) and incubated with anti-myosin heavy chain (MHC) antibody (MF20, Developmental Studies Hybridoma Bank, University of Iowa, Iowa City, IA) at 1∶200 dilution at 4°C overnight. The anti-MHC antibody was detected by incubating the cells with anti-mouse IgG FITC antibody (Sigma-Aldrich, St. Louis, MO) at 1∶200 dilution at room temperature for 1 h. Cell nuclei were stained by incubating the cells in 1 µg/ml DAPI (Sigma-Aldrich) for 1 min at room temperature. Fluorescence was detected with a Nikon eclipse E600 florescence microscope (Nikon Metrology, Inc., Brighton, MI).

### Cell proliferation assay

The numbers of viable cells at 0, 24, 48, and 72 h after initiation of culture were measured using the nonradioactive CellTiter 96 assay kit, according to the manufacturer's (Promega) instructions. The absorbance at 570 nm reflected the number of viable cells. Cell proliferation rate was represented by the slope of the line that connected the absorbance at different times in culture.

### Characterization of myotubes

To visualize myotubes and nuclei, differentiating myoblasts were washed with PBS and fixed in 4% paraformaldehyde for 10 min. Cells were then stained with Giemsa (Invitrogen) for 10 min. Total cell nuclei and nuclei within myotubes were counted using the NIH ImageJ software. A muscle cell containing 3 or more nuclei was considered as a myotube, as defined previously [17]. Fusion index was calculated as the number of nuclei in myotubes divided by the total number of nuclei counted. Average number of nuclei per myotube was determined by dividing the number of nuclei in myotubes by the total number of myotubes.

### Western blot analysis

Total cellular protein lysates were prepared and western blot analyses were conducted essentially as described previously [18], except that the primary antibodies were detected using IRDye secondary antibodies at 1∶20,000 dilution (LI-COR, Lincoln, Nebraska) and the ODYSSEY CLx system (LI-COR). The following primary antibodies were used: anti-myosin heavy chain MF20 at 1∶500 dilution (Developmental Studies Hybridoma Bank), anti-myogenin F5D at 1∶500 dilution (Developmental Studies Hybridoma Bank), and anti-β-actin N21 at 1∶1,000 dilution (Santa Cruz Biotechnology, Santa Cruz, CA).

### Statistical analyses

Two-tailed student's t test was used to determine the significance of the difference between two groups. The differences between more than two groups were analyzed by ANOVA followed by Tukey HSD multiple comparisons. These tests were performed using the JMP software (SAS, Cary, NC). A difference was considered significant when the associated *P* value was less than 0.05. All data were expressed as means ± s.e.m. (standard error of the mean).

## Results

### Stac3 knockdown promoted the differentiation of C2C12 myoblasts into myotubes

**Figure 1:**
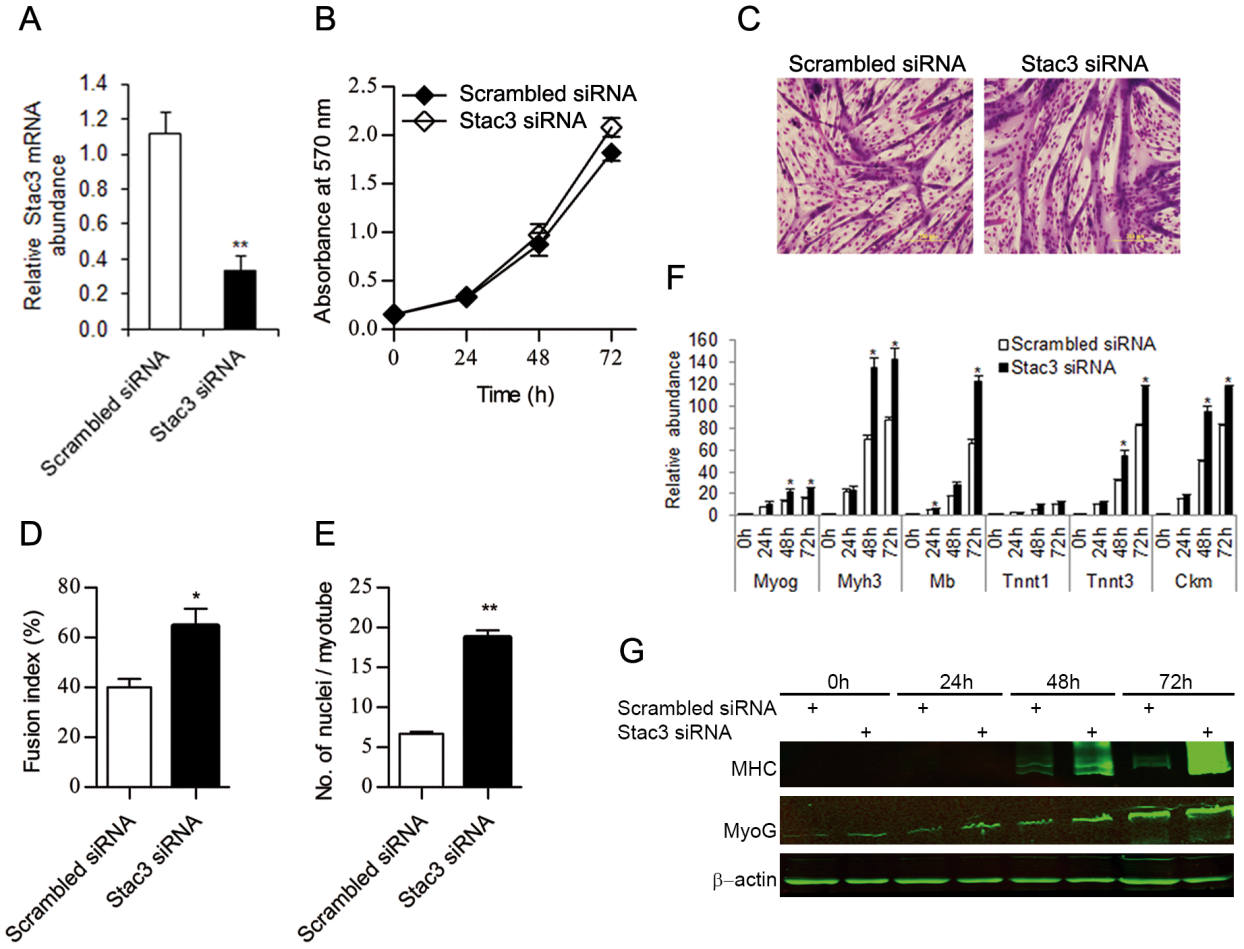
Effect of Stac3 knockdown on proliferation and differentiation of C2C12 myoblasts. C2C12 myoblasts were transfected with a pool of three Stac3 siRNAs or a scrambled siRNA and were expanded in growth medium or induced to differentiate into myotubes in differentiation medium. (**A**) Relative expression levels of Stac3 mRNA in C2C12 cells transfected with Stac3 siRNAs or scrambled siRNA at 48 h after siRNA transfection. (**B**) Proliferation rate of C2C12 cells transfected with Stac3 siRNAs or scrambled siRNA. Cell proliferation rate was estimated by measuring the numbers of viable cells at 0, 24, 48, and 72 h after transfection. The absorbance at 570 nm on the y-axis represents the number of viable cells. The proliferation rate was not different between cells transfected with Stac3 siRNAs and those with scrambled siRNA. (**C**) Representative images of C2C12 cells transfected with Stac3 siRNAs or scrambled siRNA at 72 h of differentiation. The cells were stained with Giemsa. (**D**) Fusion index at 72 h of differentiation. Fusion index was calculated as the percentage of total nuclei that resided in cells containing 3 or more nuclei. (**E**) Average number of nuclei per myotube at 72 h of differentiation. (**F**) Relative expression levels of myogenin (Myog), myosin heavy chain 3 (Myh3), myoglobin (Mb), troponin T type 1 (Tnnt1) and 3 (Tnnt3), and creatine kinase, muscle (Ckm) mRNAs at 0, 24, 48, and 72 h of differentiation. (**G**) Western blots of myosin heavy chain (MHC), myogenin (MyoG), and β-actin (loading control) proteins at 0, 24, 48, and 72 h of differentiation. Data are expressed as means ± s.e.m. (n  =  4 independent cell cultures). * *P*<0.05; ** *P*<0.01 versus “Scrambled siRNA”.

We investigated the potential role of Stac3 in myoblast proliferation by siRNA-mediated knockdown of Stac3 in C2C12 myoblasts. Transfecting these cells with a pool of three siRNAs targeting Stac3 mRNA decreased the expression of endogenous Stac3 mRNA by 65% as compared to the transfection of a scrambled siRNA (*P*<0.01, Fig. 1A). However, Stac3 siRNAs had no effect on the proliferation rate of C2C12 myoblasts (*P*>0.1, Fig. 1B). To determine the potential role of Stac3 in myoblast differentiation, C2C12 myoblasts transfected with Stac3 siRNAs or scrambled siRNA were induced to differentiate, and their differentiation status was assessed by measuring the fusion index and average number of nuclei per myotube. At 72 h of differentiation, more C2C12 myoblasts transfected with Stac3 siRNAs formed myotubes than those transfected with scrambled siRNA (66% vs. 40%, *P*<0.05; Fig. 1C and 1D). Myotubes formed from Stac3 siRNAs-transfected C2C12 myoblasts had more nuclei on average than those from scrambled siRNA-transfected C2C12 myoblasts (19 vs. 7 nuclei/myotube, *P*<0.01, Fig. 1C and 1E). These morphological data indicated that Stac3 knockdown stimulated the differentiation of C2C12 myoblasts into myotubes.

We also assessed the differentiation status of C2C12 myoblasts by measuring the mRNA levels of 6 myogenic markers including myogenin (Myog), myosin heavy chain 3 (Myh3), myoglobin (Mb), troponin T type 1 and 3 (Tnnt1 and Tnnt3), and creatine kinase, muscle (Ckm), and the protein levels of 2 myogenic markers including myogenin (MyoG) and myosin heavy chain (MHC) at 0, 24, 48, and 72 h of differentiation. The cells transfected with Stac3 siRNAs had greater expression of Myog, Myh3, Mb, Tnnt1, Tnnt3, and Ckm mRNAs than those transfected with scrambled siRNA at 24, 48, and/or 72 h of differentiation (*P*<0.05, Fig. 1F). The former cells also had greater expression of MyoG and MHC proteins than the latter at 24, 48, and/or 72 h of differentiation (Fig. 1G). These myogenic marker expression data further indicated that Stac3 knockdown promoted the differentiation of C2C12 myoblasts into myotubes.

**Figure 2:**
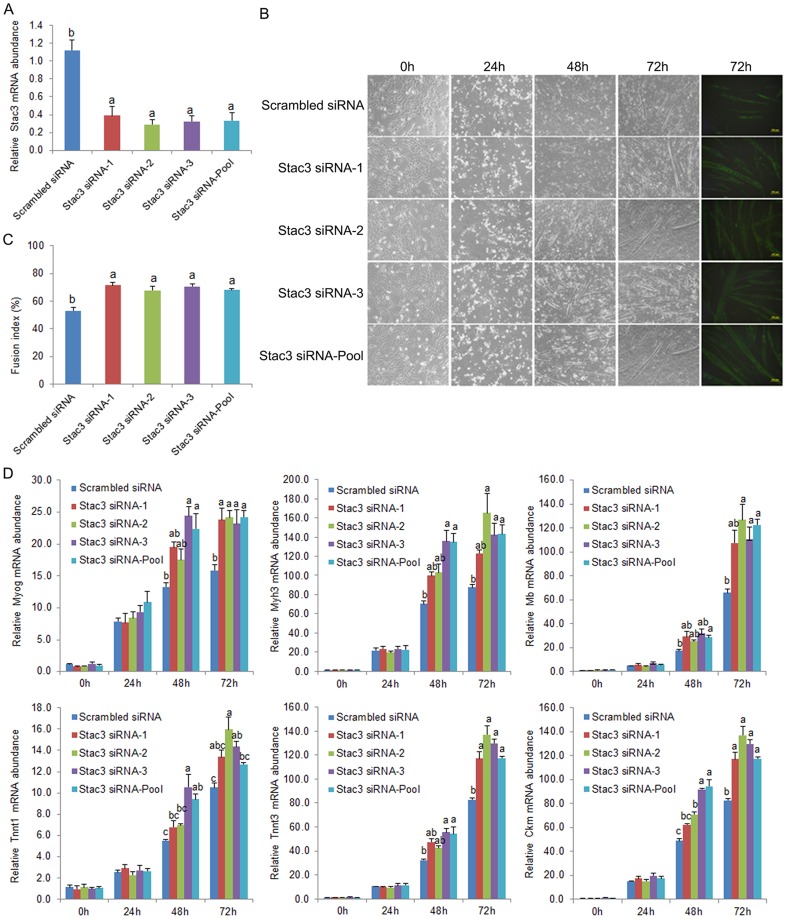
Effects of individual and pooled Stac3 siRNAs on differentiation of C2C12 myoblasts. Three Stac3 siRNAs (MSS239387, MSS239388, and MSS239389 from Invitrogen, Carlsbad, CA; indicated as Stac3 siRNA-1, -2, and -3, respectively, in the graphs) were transfected into C2C12 myoblasts separately (30 nM) or in combination (10 nM each). Transfection with 30 pmol of a scrambled siRNA (Invitrogen) was used as the control. Following transfection, the cells were maintained in growth medium or induced to differentiate into myotubes in differentiation medium. (**A**) Relative expression levels of Stac3 mRNA at 48 h after transfection. (**B**) Representative images of C2C12 cells at 0, 24, 48, and 72 h of differentiation. Cells at the far right were stained with anti-myosin heavy chain antibody (MF20, Developmental Studies Hybridoma Bank, University of Iowa, Iowa City, IA). (**C**) Fusion index at 72 h of differentiation. Fusion index was calculated as the percentage of total nuclei that resided in cells containing 3 or more nuclei. (**D**) Relative expression levels of myogenin (Myog), myosin heavy chain 3 (Myh3), myoglobin (Mb), troponin T type 1 (Tnnt1) and 3 (Tnnt3), and creatine kinase, muscle (Ckm) mRNAs at 0, 24, 48, and 72 h of differentiation. Data are expressed as means ± s.e.m. (n  =  3 independent cell cultures). Bars not labeled with the same letter are statistically different (*P*<0.05).

To validate the specificity of the effect of siRNA-mediated Stac3 knockdown on myoblast differentiation, the three Stac3 siRNAs used as a pool in the above experiments were transfected individually into C2C12 myoblasts. These transfections decreased Stac3 mRNA expression (Fig. 2A) and increased the fusion index (Fig. 2B and 2C) and mRNA expression of Myog, Myh3, Mb, Tnnt1, Tnnt3, and Ckm (Fig. 2D) in a similar manner to transfection of the three siRNAs in combination. These data ruled out the possibility that off-target effects mediated Stac3 siRNAs-enhanced differentiation of C2C12 myoblasts.

### Stac3 overexpression inhibited the differentiation of C2C12 myoblasts into myotubes

**Figure 3:**
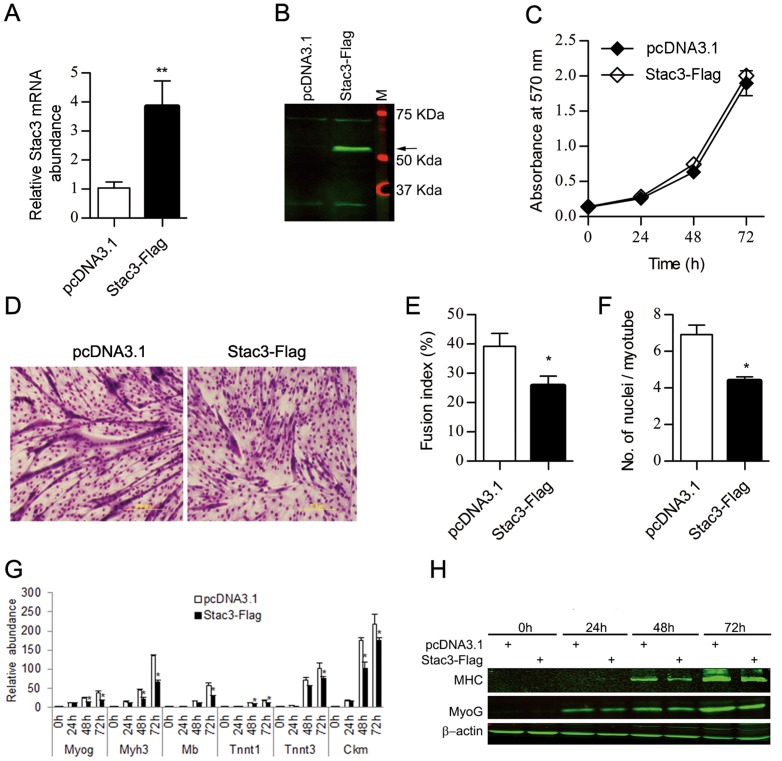
Effect of Stac3 overexpression on proliferation and differentiation of C2C12 myoblasts. C2C12 myoblasts were transfected with the Stac3-Flag fusion protein expression plasmid pcDNA3.1/Stac3-Flag or empty vector pcDNA3.1, and were expanded in growth medium or induced to differentiate into myotubes in differentiation medium. (**A**) Relative expression levels of Stac3 mRNA in C2C12 cells at 48 h after transfection. (B) Expression levels of Stac3-Flag fusion protein in C2C12 cells at 48 h after transfection. The Stac3-Flag fusion protein was detected by anti-Flag antibody and indicated by an arrow. (**C**) Proliferation rate of C2C12 cells transfected with pcDNA3.1/Stac3-Flag or pcDNA3.1. The proliferation rate was not different between cells transfected with pcDNA3.1/Stac3-Flag and pcDNA3.1. (**D**) Representative images of transfected C2C12 cells at 72 h of differentiation. (**E**) Fusion index of transfected C2C12 cells at 72 h of differentiation. (**F**) Average number of nuclei per myotube at 72 h of differentiation. (**G**) Relative expression levels of myogenin (Myog), myosin heavy chain 3 (Myh3), myoglobin (Mb), troponin T type 1 (Tnnt1) and 3 (Tnnt3), and creatine kinase, muscle (Ckm) mRNAs at 0, 24, 48, and 72 h of differentiation. (**H**) Western blots of myosin heavy chain (MHC), myogenin (MyoG), and β-actin (loading control) proteins at 0, 24, 48, and 72 h of differentiation. Data are expressed as means ± s.e.m. (n  =  4 independent cell cultures). * *P*<0.05; ** *P*<0.01 versus “pcDNA3.1”.

We also determined the effect of Stac3 overexpression on proliferation and differentiation of C2C12 myoblasts. We achieved Stac3 overexpression by transfecting the cells with a Stac3-Flag fusion protein expression plasmid and confirmed Stac3 overexpression at both the mRNA (Fig. 3A) and protein (Fig. 3B) levels. Stac3 overexpression did not alter the proliferation rate of C2C12 myoblasts (*P*>0.1, Fig. 3C). At 72 h of differentiation, approximately 25% of C2C12 myoblasts transfected with the Stac3-Flag expression plasmid formed myotubes, whereas this percentage was nearly 40% for those transfected with the empty vector (*P*<0.05, Fig. 3D and 3E). Myotubes formed from Stac3-overexpressing C2C12 myoblasts had 4 nuclei on average, whereas those formed from control cells had 7 nuclei (*P*<0.05, Fig. 3D and 3F). At 48 and 72 h of differentiation, myotubes formed from Stac3-overexpressing C2C12 myoblasts had less expression of Myog, Myh3, Mb, Tnnt1, Tnnt3, and Ckm mRNAs than those formed from C2C12 myoblasts transfected with the empty vector (*P*<0.05, Fig. 3G). The former cells also expressed less myogenin protein than the latter at 24, 48, and 72 h of differentiation, and less myosin heavy chain protein than the latter at 48 and 72 h of differentiation (Fig. 2H). These changes caused by Stac3 overexpression were opposite to those caused by Stac3 knockdown in C2C12 myoblasts (Fig. 1).

### Myoblasts from Stac3 mutant mouse embryos showed accelerated differentiation in culture

**Figure 4:**
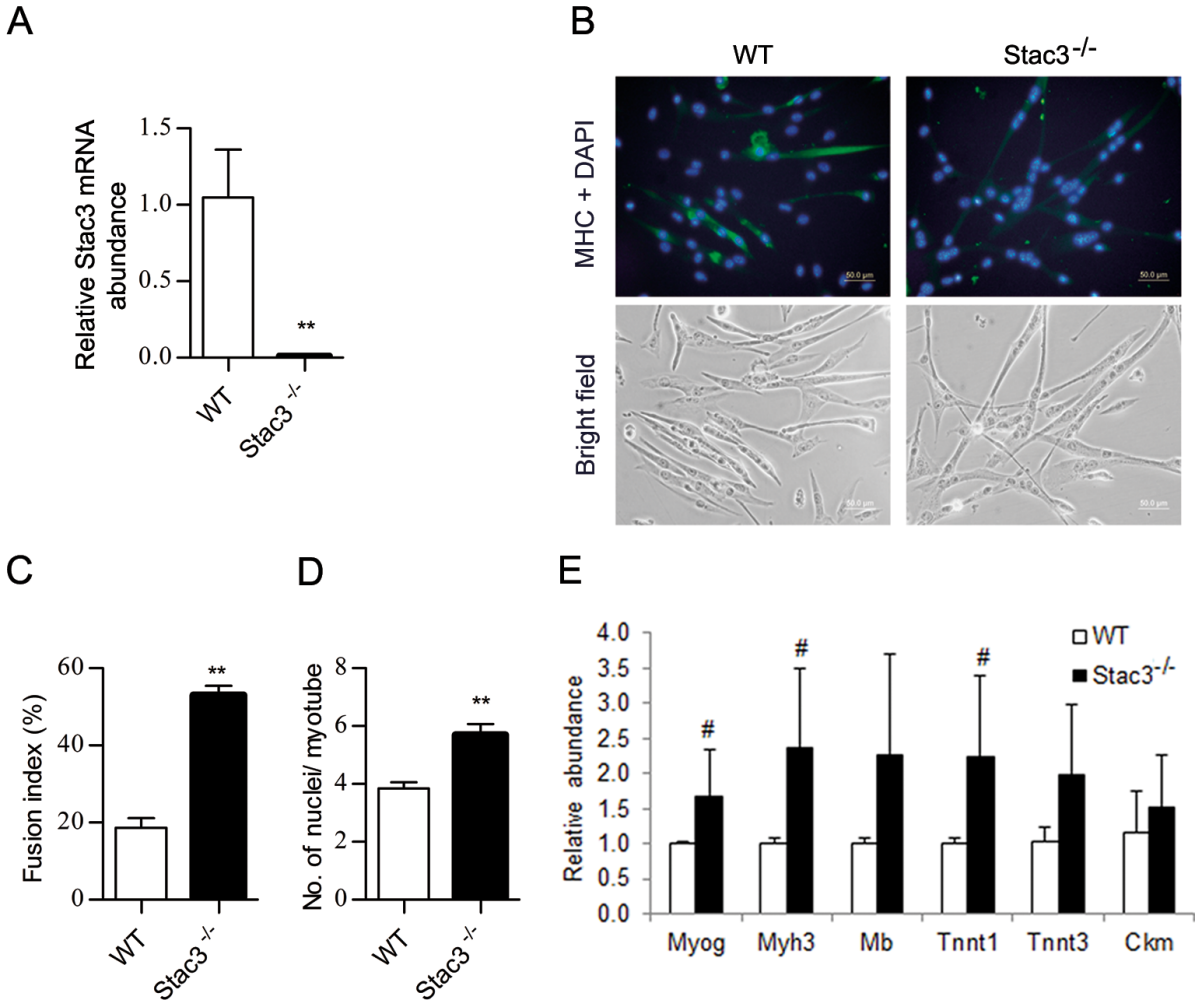
Effect of Stac3 knockout on differentiation of embryonic mouse myoblasts. Myoblasts from limb muscles of E17.5 Stac3 homozygous mutant (Stac3^-/-^) or wild-type (WT) mouse fetuses were isolated, expanded and enriched in growth medium, and induced to differentiate into myotubes in differentiation medium. (**A**) Quantitative RT-PCR analysis of Stac3 mRNA in Stac3 mutant and wild-type myoblasts. (**B**) Representative images of Stac3 mutant and wild-type myoblasts at 72 h of differentiation. Myotubes were stained with anti-myosin heavy chain (MHC) antibody (green), and cell nuclei with DAPI (blue). (**C**) Fusion index at 72 h of differentiation. (**D**) Average number of nuclei per myotube at 72 h of differentiation. (**E**) Relative expression levels of myogenin (Myog), myosin heavy chain 3 (Myh3), myoglobin (Mb), troponin T type 1 (Tnnt1) and 3 (Tnnt3), and creatine kinase, muscle (Ckm) mRNAs at 72 h of differentiation. Data are expressed as means ± s.e.m. (n  =  2 or 3 independent cell cultures). ** *P*<0.01; ^#^ *P = 0.2* versus “WT”.

We isolated myoblasts from E17.5 Stac3 homozygous mutant mouse embryos and wild-type littermates and compared their potential to differentiate into myotubes in culture. Quantitative RT-PCR analysis confirmed the absence of Stac3 mRNA in myoblasts from Stac3 homozygous mutant embryos (Fig. 4A). At 72 h of differentiation, 53% of Stac3 mutant myoblasts fused into myotubes, whereas this percentage was much lower (18%) for wild-type myoblasts (*P*<0.01, Fig. 4B and 4C). On average, myotubes formed from Stac3 mutant myoblasts contained 2 more nuclei than those from wild-type myoblasts (*P*<0.01, Fig. 4B and 4D). These morphological differences indicated that Stac3 mutant myoblasts differentiated more rapidly than wild-type myoblasts in culture. At 72 h of differentiation, Stac3 mutant myoblasts expressed 50 to 150% more Myog, Myh3, Mb, Tnnt1, and Tnnt3 mRNAs than wild-type myoblasts (*P* = 0.2 to 0.3; Fig. 4E).

## Discussion

In this study we determined whether Stac3 plays a role in myoblast proliferation and differentiation in two myogenic cell systems: C2C12 myoblast line and primary murine myoblasts. While Stac3 knockdown had no effect on proliferation of C2C12 myoblasts, it stimulated their differentiation into myotubes, as evidenced by increased fusion rate, increased average number of nuclei per myotube, and increased mRNA and protein expression of myogenic markers including myogenin, the myogenic regulatory factor that controls the terminal differentiation of myoblasts into myotubes [4], [7]. In contrast, Stac3 overexpression inhibited the differentiation of C2C12 myoblasts into myotubes, as evidenced by decreased fusion rate, decreased average number of nuclei per myotube, and decreased mRNA and protein expression of myogenic markers. Judged by the number and size of myotubes formed, myoblasts from Stac3-null mouse embryos differentiated more rapidly in culture than wild-type myoblasts. These results imply an inhibitory role of Stac3 in myoblast differentiation into myotubes.

Our finding that Stac3 inhibits C2C12 t differentiation into myotubes is seemingly at odds with an earlier report concluding that Stac3 is essential for myotube formation from C2C12 cells [14]. The cause for this discrepancy is not clear. It could be due to the different culture conditions or different passage numbers of C2C12 cells, which are difficult to control, used between the two studies. However, an essential role of Stac3 for myotube formation, as concluded in the earlier study [14], is not supported by the phenotypes of Stac3 knockout mice and Stac3 knockdown zebrafish, where myofibers clearly are formed [11]–[14]. An inhibitory role of Stac3 in myoblast differentiation into myotubes indeed can explain some of the phenotypes in Stac3 knockout mice. Skeletal muscle of Stac3 knockout mice had fewer yet larger myofibers and had a disproportional number of myofibers with large cross-sectional areas [13]. In theory, the increased size in myofibers can be caused either by hastened myoblast differentiation and fusion into myotubes or by hypertrophy of fused myotubes. However, it is unlikely that Stac3 mutant myotubes were undergoing hypertrophy because they indeed had fewer and smaller myofibrils than wild-type or Stac3 heterozygous mutant myotubes [13]. The relative number of proliferating myoblasts versus differentiating myoblasts during muscle development determines the total number of myofibers formed in mature muscle [19], [20]. If myoblasts withdraw from the cell cycle and begin differentiation before an adequate number of founder myoblasts have developed, this will decrease the overall number of myofibers formed. Therefore, hastened myoblast differentiation and fusion into myotubes could also explain why Stac3 knockout skeletal muscle had fewer total myofibers than wild-type or Stac3 heterozygous mutant skeletal muscle [13].

In summary, the present study suggests that, in myoblasts, Stac3 plays a role in inhibiting the differentiation of these cells into myotubes. Myogenesis is a tightly controlled program; premature myoblast differentiation would lead to the formation of dysfunctional myofibers [21], [22]. Thus, Stac3 could be one of those factors that prevent precocious myoblast differentiation during skeletal muscle development. Obviously, the mechanism by which Stac3 inhibits myoblast differentiation into myotubes is an interesting future question to investigate. The SH3 domain contained in the Stac3 protein is a classical protein-protein interaction domain [23], [24]; therefore, Stac3 might inhibit myoblast differentiation through interaction with other proteins. It would be interesting to know if Stac3 interacts with or affects the expression of proteins such as Delta-like protein 1, myogenin, MRF4 (Myf6), Stat1, and Stat3 that are known to play a role in normal or premature myoblast differentiation [21], [22], [25].
