# Correction: Stac3 Inhibits Myoblast Differentiation into Myotubes

**DOI:** 10.1371/journal.pone.0105240

**Published:** 2014-08-07

**Authors:** The PLOS ONE Staff

There are errors in Figure 2. Please see the corrected Figure 2 here.

**Figure 2:**
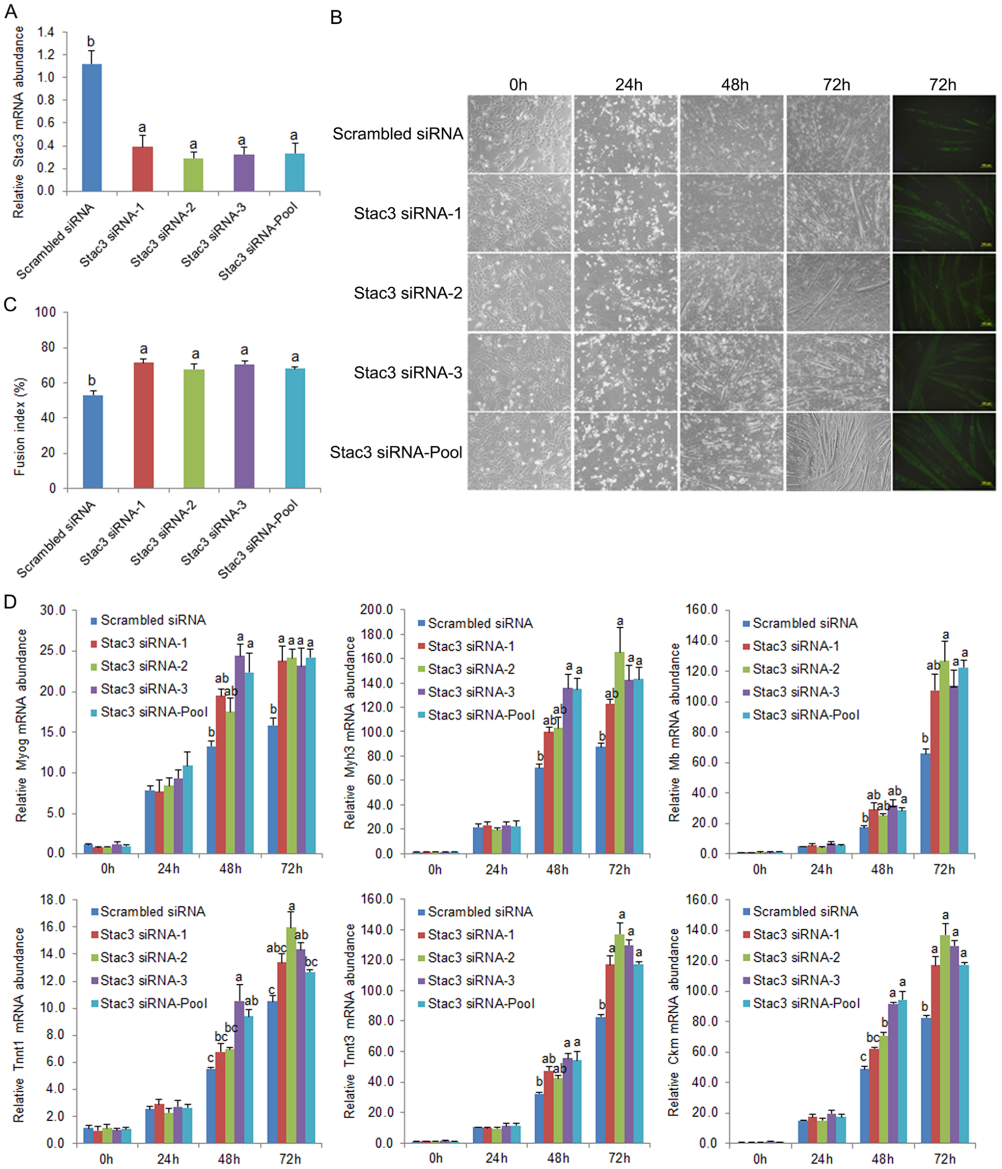
Effects of individual and pooled Stac3 siRNAs on differentiation of C2C12 myoblasts. Three Stac3 siRNAs (MSS239387, MSS239388, and MSS239389 from Invitrogen, Carlsbad, CA; indicated as Stac3 siRNA-1, -2, and -3, respectively, in the graphs) were transfected into C2C12 myoblasts separately (30 nM) or in combination (10 nM each). Transfection with 30 pmol of a scrambled siRNA (Invitrogen) was used as the control. Following transfection, the cells were maintained in growth medium or induced to differentiate into myotubes in differentiation medium. (A) Relative expression levels of Stac3 mRNA at 48 h after transfection. (B) Representative images of C2C12 cells at 0, 24, 48, and 72 h of differentiation. Cells at the far right were stained with anti-myosin heavy chain antibody (MF20, Developmental Studies Hybridoma Bank, University of Iowa, Iowa City, IA). (C) Fusion index at 72 h of differentiation. Fusion index was calculated as the percentage of total nuclei that resided in cells containing 3 or more nuclei. (D) Relative expression levels of myogenin (Myog), myosin heavy chain 3 (Myh3), myoglobin (Mb), troponin T type 1 (Tnnt1) and 3 (Tnnt3), and creatine kinase, muscle (Ckm) mRNAs at 0, 24, 48, and 72 h of differentiation. Data are expressed as means ± s.e.m. (n  =  3 independent cell cultures). Bars not labeled with the same letter are statistically different (P<0.05).
